# Prediction of Druggable Proteins Using Machine Learning and Systems Biology: A Mini-Review

**DOI:** 10.3389/fphys.2015.00366

**Published:** 2015-12-08

**Authors:** Gaurav Kandoi, Marcio L. Acencio, Ney Lemke

**Affiliations:** ^1^Department of Electrical and Computer Engineering, Iowa State UniversityAmes, IA, USA; ^2^Department of Physics and Biophysics, Institute of Biosciences of Botucatu, UNESP – São Paulo State UniversityBotucatu, Brazil

**Keywords:** druggability, machine learning, systems biology, review, drug targets, sequence properties, structural properties, network topology

## Abstract

The emergence of -omics technologies has allowed the collection of vast amounts of data on biological systems. Although, the pace of such collection has been exponential, the impact of these data remains small on many critical biomedical applications such as drug development. Limited resources, high costs, and low hit-to-lead ratio have led researchers to search for more cost effective methodologies. A possible alternative is to incorporate computational methods of potential drug target prediction early during drug discovery workflow. Computational methods based on systems approaches have the advantage of taking into account the global properties of a molecule not limited to its sequence, structure or function. Machine learning techniques are powerful tools that can extract relevant information from massive and noisy data sets. In recent years the scientific community has explored the combined power of these fields to propose increasingly accurate and low cost methods to propose interesting drug targets. In this mini-review, we describe promising approaches based on the simultaneous use of systems biology and machine learning to access gene and protein druggability. Moreover, we discuss the state-of-the-art of this emerging and interdisciplinary field, discussing data sources, algorithms and the performance of the different methodologies. Finally, we indicate interesting avenues of research and some remaining open challenges.

## Introduction

Biological systems are complex and the response to a chemical substance is often unpredictable. When a chemical substance, like a drug, interferes with the natural biology of a system, the effect is usually undesirable. Pharmaceutical industry has come a long way when it comes to drug discovery. Rapid advancement in the technology over the years and the increasing understanding of biology has led to designing drugs more efficiently. While the approved drugs increased over the past decade, they did not match the increase in cost of drug development (Csermely et al., 2013).

Druggability is the property of a druggable molecule (i.e., a biological target) by virtue of which it elicits a favorable clinical response when it contacts a drug-like compound. While the majority of druggable targets today are proteins, nucleic acids are slowly replacing them (Imming et al., 2006; Davidson and McCray, 2011). According to Gashaw et al. (2011), an ideal drug target should have the following properties: favorable assayability for high throughput screening, capacity to modify a disease, low impact on the modulation of physiological conditions or other diseases, differential expression across the body for specific targeting, the existence of a biomarker to monitor its efficacy and freedom to operate, i.e., lack of competitive binding.

Experimentally evaluating all proteins or nucleic acid fragments for their druggability is a daunting task. Our lack of knowledge about the biology of disease at molecular level further complicates the situation. With these uncertainties at hand, our sample space for a potential drug target is enormous. It is therefore impractical to clinically evaluate all drug targets before being able to first prioritize them. Due to these facts, computational models that can predict drug targets with high sensitivity while maintaining a high specificity on a genome-wide scale would be highly welcomed.

With the advancements in technology, we now have access to a plethora of data including protein-protein interaction (PPI), metabolic and gene regulatory networks, protein and gene expression profiles, and other system-level data. Although consolidating these diverse data sets is still challenging, progress has been made in the past few years. It is now possible to combine these system-level data with data mining tools like machine learning to build predictive models. Such analyses have the potential of identifying biologically relevant patterns that confer druggability to potential drug targets (Costa et al., 2010).

In this mini-review, we discuss the current state of machine learning-based methods for druggability prediction, specifically those using system-level features. Despite the importance of this subject, to the best of our knowledge, only seven papers using machine learning approaches based on system-level data to predict druggable proteins and genes have been published so far (Table 1; Yao and Rzhetsky, 2008; Zhu et al., 2009; Costa et al., 2010; Emig et al., 2013; Laenen et al., 2013; Jeon et al., 2014; Li et al., 2015).

**Table 1:** **Summary of the papers analyzed in this mini-review**.

**References**	**Learning instances**	**Learning features**		**Machine learning algorithms**	**Prediction performance metrics**	**Results**
		**Source**	**Type of Feature**			
Zhu et al., 2009	DrugBank	BioGRID	Connectivity degree, cluster coefficient, distance-based measures, topological coefficient	Support Vector Machine	AUC	AUC: 69.21%
Jeon et al., 2014	DrugBank, Therapeutics Target Database	Bossi and Lehner, 2009	GARP score, RMA intensity, row chromosomal copy number, mutation occurrence and closeness centrality (combined or isolated)	SVM-recursive feature elimination (SVM-REF) method for feature selection; SVM-RBF kernels for predictions	Accuracy, Specificity, AUC	Avg. accuracy: 91.69% Avg. specificity: 91.91% Avg. AUC: 78% (combined)
Li et al., 2015	DrugBank	HIPPIE	Combination of various network distance-based measures and sequence features of proteins	Random Forest with minimum Redundancy Maximum Relevance (mRMR) Feature Selection	Accuracy, Sensitivity, Specificity, Precision, Matthews correlation coefficient	Accuracy: 87.05% Sensitivity: 90.28% Specificity: 83.83% Precision: 84.82% Matthews correlation coefficient: 0.7427 (Avg. of 10 random samples)
Laenen et al., 2013	PubChem, ChEMBL and BindingDB	STRING, GEO (Edgar et al., 2002)	Combination of kernel and correlation diffusion and differential gene expression	Rank-based method	AUC	Kernel: 76–91% Correlation: 89–92%
Emig et al., 2013	Integrity	metaBase (Bureeva et al., 2009), GEO (Edgar et al., 2002)	Combination of neighborhood scoring, interconnectivity, network propagation, random walk and differential gene expression	Logistic regression model	AUC	AUC: 63.27–93.19%
Yao and Rzhetsky, 2008	DrugBank	HPRD	Combination of connectivity, betweenness, tissue expression entropy, constant corrected ratio of non-synonymous and synonymous mutations and functional family assignment	Naive Bayesian, logistic regression, radial basis function network, Bayesian networks	AUC	Naive Bayes: 70.43% Logistic regression: 72.57% RBF network: 60.93% Bayesian Network: 72.31%
Costa et al., 2010	Yildirim et al., 2007	BioGRID, DIP, HPRD, IntAct, MINT, MIPS-MPPI, TRED, human metabolic model Recon 1	Combination of several network measures, tissue expression profile and subcellular localization	Decision tree-based meta-classifier	AUC, Recall, Precision	AUC: 82% Recall: 78.2% Precision: 74.8%

Usually, the development of predictive models in a machine learning approach is accomplished by the following steps: selection of learning instances (in this case, the druggable and non-druggable molecules) and attributes (in this case, system-level features), selection of learning algorithms and evaluation of the predictive performance of models. We structured this mini-review according to these steps: first we discuss the learning instances, then the attributes related to the system-level-based prediction of druggability with their performance metrics and finally we discuss the most used machine learning algorithms in this field. Most of the discussions are based on the papers shown in Table 1. Some of the common terminologies used in this mini-review are described in Table 2.

**Table 2:** **Brief description about the common terminologies used in this mini-review**.

**Concept**	**Description**
Druggability	The property of a druggable molecule (i.e., a biological target) by virtue of which it elicits a favorable clinical response when it contacts a drug-like compound
Systems Biology	Study of the complex biological systems using mathematical and computational modeling
Machine Learning	Subfield of computer science devoted to the development and utilization of algorithms that can learn from and make predictions on data
Network Measures	Numerical attributes used to describe the role and position of every node in a network
Ensemble algorithms	Collection of machine learning algorithms in which the final consensus prediction is made using results from each component algorithm
Support Vector Machines (SVM)	A model that takes the input training data and maps the data points in space and then tries to find a hyperplane that can be used to distinctly classify the data into their respective classes
Decision Tree	Machine learning algorithms based on decision support tools that make use of a graph of conditions and their possible consequences
Random Forest	Ensemble learning algorithm that combines results from multiple decision trees and output the consensus predictions
Closeness Centrality	Network measure that indicates how close each node is to every other node in the network
Betweenness Centrality	Fraction of shortest paths between all nodes passing through the given node

## Learning instances: Druggable and non-druggable proteins

It is critical to efficiently store information pertaining to drugs and their targets, i.e., druggable molecules. There is an abundance of biochemical data available in the literature that can be used to formulate hypotheses about how a phenotypic condition can be targeted. According to the Pathguide, a pathway resource list that contains information about hundreds of biological databases dedicated to molecular interaction (Bader et al., 2006), several resources specific to drugs and drug targets have been developed to help address this issue. Among these resources, the following were used in the papers commented in this mini-review: DrugBank (Knox et al., 2011), Therapeutic Target Database (TTD, Chen et al., 2002), ChEMBL (Bento et al., 2014), PubChem (Bolton et al., 2008), BindingDB (Liu et al., 2007), and Integrity^1^. From the Table 1 it is clear that DrugBank has been the most popular database among the investigators willing to predict druggable proteins based on system-level data: of the seven papers discussed in this mini-review, four used drug-target data from DrugBank (Yao and Rzhetsky, 2008; Zhu et al., 2009; Jeon et al., 2014; Li et al., 2015).

Interestingly, the preference for DrugBank as a source of drug-protein interactions among the dozens of databases dedicated to the storage of this type of data is not clearly explained in the papers discussed here. The fact that most of the data in DrugBank are expertly curated from primary literature sources would be the reason that makes this database so popular. However, all other drug-protein interactions databases cited in this mini-review are similar to DrugBank in this sense. So, one possible explanation for the popularity of DrugBank is that, in comparison to other databases, its collection of drug-protein interactions can be easily obtained.

Even with the presence of high-quality data and completeness of above-mentioned databases, they lack quantitative information about the binding affinity that could be used to evaluate the reliability of the interactions, except for the BindingDb that reports some of these quantitative measures (Liu et al., 2007). Ideally, the prediction of druggable proteins would be more realistic if interaction affinities measured by bioactivity assays were taken in consideration. As none of the studies analyzed here report the utilization of quantitative features to construct prediction models of druggable proteins, so the prediction performances reported in Table 1 are likely to be overoptimistic due to the oversimplified formulation of the drug–target prediction problem as a binary problem (Pahikkala et al., 2014).

## Learning attributes and prediction performance

Many different attributes have been used to generate models able to predict druggability such as sequence and structural properties (Li and Lai, 2007; Bakheet and Doig, 2009; Fauman et al., 2011). Here we focus solely on system-level properties like topological features of networks and gene expression profile.

To calculate the topological features of networks (henceforth called network measures) to be used as learning features in a machine learning approach, first it is necessary to build the PPI networks from which these measures are calculated. PPIs can be obtained from a multitude of databases, such as String (Jensen et al., 2009), Human Integrated Protein-Protein Interaction rEference (HIPPIE; Schaefer et al., 2012), BioGrid (Breitkreutz et al., 2008), and Human Protein Reference Database (HPRD; Peri et al., 2004), among others. Different from the drug-target-dedicated databases in which DrugBank is the preferred database, there is no preferred PPI database among investigators involved in the prediction of druggable proteins by system-level data-based machine learning approaches as can be seen in Table 1. This reflects on the need to develop a standardized resource that can harbor PPI information, similar to DrugBank for drug-targets. The IntAct (Orchard et al., 2014), an open-source, open data molecular interaction database populated by data either curated from the literature or from direct data depositions, for example, is one of the promising initiatives in this regard.

The immediate consequence of the utilization of different PPIs databases in the different studies is the inability to compare the prediction performances of the models constructed in these studies: from different PPI networks, distinct values for network measures are obviously obtained. Moreover, in addition to the oversimplification of the drug-target interactions problem as discussed above, it is also worth to mention that all prediction performances shown in Table 1 should be cautiously considered as PPIs used to construct the networks are biased toward well-studied genes and proteins despite the fact that the PPI databases provide hundreds of thousands of interactions. Recent studies on the construction of interactomes are however believed to better capture unbiased molecular interactions (Rolland et al., 2014).

Regardless of the constraints discussed above, we analyze and compare here the prediction performances of the models based on network measures alone or in combination with gene expression data. We cannot determine how accurate these comparisons are, but at least they can indicate trends toward the predictability of druggability by these learning attributes.

Researchers sought to investigate whether druggable proteins occupy certain regions in a PPI network—thus implying network measures distinct from other proteins—since, many studies had already been demonstrated that disease and essential proteins occupy specific regions in a network and, as a consequence, exhibit network measures distinct from other proteins. In fact, as observed for essential and disease proteins, druggable proteins seem to be located in specific regions in a PPI network. Yildirim et al. (2007), in their pioneering study on drug-target network and, later on, Yao and Rzhetsky (2008) and Jeon et al. (2014), found that druggable proteins show some network measures significantly different from other proteins in the PPI network.

Hence, network measures could also be potential predictors of druggability in machine learning approaches in the same way that they have been demonstrated to be potential predictors of essential and disease genes. Indeed, as shown in Table 1, machine learning approaches based on a variety of combinations of network topological features seem to be promising for predicting druggable proteins and genes. Prediction models constructed based solely on network measures achieved values of area under the receiver operating characteristic curve (AUC) of 69.21% and ~68% as demonstrated, respectively, by Zhu et al. (2009) and Jeon et al. (2014). On integration of genomic properties like GARP score, RMA intensity, row chromosomal copy number, and mutation occurrence to closeness centrality, Jeon et al. (2014) were able to improve the AUC to 78%. These figures suggest that network measures alone are moderately predictive of druggable proteins. However, more comprehensive studies in which network measures are individually and collectively used as learning attributes will be required to measure the level of predictability of druggable proteins by network measures.

Although, the other papers commented here report the creation of prediction models based on various and diverse network measures, it is not possible to evaluate the prediction performance of druggable proteins by considering only network measures since in these models they were combined with other features, mostly being gene expression profile, as shown below.

Using connectivity and betweenness in addition to other systems-level properties, including gene expression profile, Yao and Rzhetsky (2008) achieved AUCs 60–72% using different machine learning algorithms. The prediction models of Costa et al. (2010), based on various network measures, gene expression profile and subcellular localization, achieved a median AUC of 82% while correctly recovering 78.2% of known targets with a precision of 74.8%. Upon analysis of the features important to discern druggable from non-druggable genes, they found that genes encoding proteins located centrally in a transcriptional regulatory network are more probable of being a drug target. The centrally located genes were found by calculating the betweenness centrality of all genes within the transcriptional regulatory network. While all studies use different network topological features, there is an indication that drug-targets are better connected and centrally located than an average gene.

Other papers commented here also showed that the global expression profile of genes along with network measures can be potential predictors of druggability in a machine learning approach. Emig et al. (2013) achieved median AUCs in the range of 63–93% using gene expression signatures for 30 diseases along with random walk, interconnectivity, network propagation and neighborhood scoring. Laenen et al. (2013) evaluated their methods by means of assessing the AUC from predictions on 235 gene expression datasets. Using only the gene expression data, they obtained AUC in the range 64–66%. However, the combination of these expression data with network measures improved the prediction performance: while the combination of expression data with kernel diffusion achieved AUC in the range of 76–91%, the combination with the correlation diffusion method achieved AUC in the range of 89–92%.

The study conducted by Li et al. (2015) is a special case to be analyzed since they combined network and sequence features of proteins to construct predictors of druggable proteins. In spite of the fact that the process of integration between network and sequence features was not clearly showed in this paper, the constructed predictor based on eight different types of network distance-based measures obtained a sensitivity of ~90% and a precision of ~85%. According to authors, the influence of sequence features on this high prediction performance is negligible due to the low amount of sequence features among all used learning features, but this is still a matter of debate.

Taken together, despite all limitations concerning the databases of drug-protein interactions and the construction of PPI networks as previously discussed, the findings reported in these papers indicate the importance of integrating other types of systems-level data to network measures to improve the prediction of druggable proteins. It seems that only network measures are not enough to distinguish druggable from non-druggable proteins, although a large-scale study for evaluating how well-druggable proteins can be predicted solely by network measures is necessary to confirm this moderate prediction performance as previously discussed.

## Machine learning algorithms

The advent of machine learning algorithms has furthered the field of drug discovery. There are many different types of machine learning algorithms that have been used to distinguish the specific properties of two or more functional classes (druggable vs. non-druggable; enzyme vs. non-enzyme etc.) as shown in Table 1. Algorithms based on SVMs, decision trees, ensemble of classifiers, logistic regression, radial basis function, and Bayesian networks have been commonly used.

Zhu et al. (2009) and Jeon et al. (2014) both used SVM to construct their prediction model. SVMs are a set of models that maps the data points in space and then constructs a hyperplane that can be used for classification. The larger the distance of the hyperplane from the nearest data-point, better the model is. Li et al. (2015) and Costa et al. (2010) used decision-tree based ensemble algorithms. Decision trees are simple, yet powerful way to perform classification. They use decisions tree as a predictive model for classifying an object (a gene in this case) into its target class (druggable/non-druggable). The ensemble algorithms used by Li et al. (2015; Random Forest) and Costa et al. (2010; decision-tree based meta classifier) combine the prediction of multiple decision trees. The results from individual trees are combined by means of a voting strategy to produce higher confidence predictions.

Logistic regression was used by Emig et al. (2013) and Yao and Rzhetsky (2008) for their predictive modeling studies. Besides logistic regression, Yao and Rzhetsky (2008) also used other three classifiers (Bayesian network, naïve Bayes, and RBF network). Finally, Laenen et al. (2013) used a ranking method instead of an explicit machine learning algorithm to prioritize druggable proteins.

## Discussion

Drug development is a long, expensive and laborious process with a very low success rate. It is therefore critical to ensure high confidence of each step. Identifying a potential target is amongst the most preliminary stages and is therefore a necessity to ensure success during later stages. In the past few years we have seen a shift of pharmaceutical industry to employ computational prediction models early during the process.

With the explosion of high quality “omics” data and improvements in computational efficiency, large number of prediction methods has been proposed for target prioritization. Machine learning algorithms constitute the major proportion of such strategies. These methods have tried to capture the characteristics of successful drug targets to identify new targets with similar properties. Among the most commonly used features include sequence properties, role in biological networks, structural properties, gene expression profiles, and subcellular locations.

The most fundamental sequence property used for any protein function prediction is its sequence composition. Composition of the 20 amino acids has been repetitively used for predicting potential drug targets. Other commonly used properties derived from sequence include physicochemical properties like hydrophobicity, polarity, solvent accessibility, and charge etc. Structure based methods make use of the information taken from protein structures. Some commonly used structure derived properties include the characteristics of surface area, binding-sites and cavities, pockets, and volume etc.

Several prediction methods identify potential drug targets; however, they suffer limitations already known. Sequence properties alone are unable to capture the global information of a protein target and do not take into account its functional role. While the sequence can be used to predict the functional domains, it gives no information regarding the accessibility of these domains to a drug, gene expression level and its importance in the interactome. Targeting an otherwise potential target may have undesirable impact on its functional neighbors. Structural methods suffer from the sparsity of information in protein data bank (PDB). Functional networks and expression profiles are dynamic and prone to changes across conditions.

While the improvement in current technology will help better capture the global properties of all proteins, establishing data standards will be critical for evaluating diverse prediction methods.

## Future directions

Given these limitations and strengths of the current methods and the incomplete and unbalanced nature of data sets on target druggability, next generation of methods should utilize the vast biological information regarding role in functional networks, expression profiles, subcellular locations, and quantitative features of drug-protein interactions with ensemble methods in machine learning approaches to capture a more universal view of a potential target. Advances in both functional and structural genomics along with improvements in computational algorithms are a key to developing more accurate methods for target identification.

### Conflict of interest statement

The authors declare that the research was conducted in the absence of any commercial or financial relationships that could be construed as a potential conflict of interest.

## Footnotes

^1^http://integrity.thomson-pharma.com.
